# Why to account for finite sites in population genetic studies and how to do this with Jaatha 2.0

**DOI:** 10.1002/ece3.722

**Published:** 2013-09-04

**Authors:** Lisha A Mathew, Paul R Staab, Laura E Rose, Dirk Metzler

**Affiliations:** 1 Life Sciences, École Polytechnique Fédérale de LausanneLausanne, Switzerland; 2 Swiss Institiute of Bioinformatics (SIB)Lausanne, Switzerland; 3 Department of Biology, Ludwig-Maximilians-Universität MünchenPlanegg-Martinsried, Germany; 4 Institute of Population Genetics, Heinrich Heine UniversityDüsseldorf, Germany

**Keywords:** Demography estimation, infinite-sites model, model violation, mutation rate heterogeneity, next-generation sequencing

## Abstract

With the advent of next-generation sequencing technologies, large data sets of several thousand loci from multiple conspecific individuals are available. Such data sets should make it possible to obtain accurate estimates of population genetic parameters, even for complex models of population history. In the analyses of large data sets, it is difficult to consider finite-sites mutation models (FSMs). Here, we use extensive simulations to demonstrate that the inclusion of FSMs is necessary to avoid severe biases in the estimation of the population mutation rate *θ*, population divergence times, and migration rates. We present a new version of Jaatha, an efficient composite-likelihood method for estimating demographic parameters from population genetic data and evaluate the usefulness of Jaatha in two biological examples. For the first application, we infer the speciation process of two wild tomato species, *Solanum chilense* and *Solanum peruvianum*. In our second application example, we demonstrate that Jaatha is readily applicable to NGS data by analyzing genome-wide data from two southern European populations of *Arabidopsis thaliana*. Jaatha is now freely available as an R package from the Comprehensive R Archive Network (CRAN).

## Introduction

In recent years, a great number of reports on whole-genome data sets have followed the advent of next-generation sequencing (NGS) technologies (e.g., pyrosequencing, Margulies et al. 2005). Examples are the introduction of the human 1000 genomes project (1000 Genomes Project Consortium 2010) and the 1001 genomes project of *Arabidopsis thaliana* (Weigel and Mott 2009; Cao et al. 2011). Though less extensive in number of genomes, sequenced whole-genome data are available from several other organisms, including *Drosophila* (Begun et al. 2007), mouse (Keane et al. 2011), and *Escherichia coli* (Lukjancenko et al. 2010).

The available vast amounts of data enable us to estimate parameters of complex models with greater precision (Lascoux and Petit 2010; Keinan and Clark 2012). These models accommodate the biological information relevant to the study organism to shed light on evolutionary processes, such as speciation (The Heliconius Genome Consortium 2012). Furthermore, detailed models can be prerequisites for inferring natural selection (e.g., Clotault et al. 2012). The necessity to account for demography first was pointed out due to its “selection-mimicking” effects on genetic variability (Robertson 1975; Andolfatto and Przeworski 2000; Teshima et al. 2006; Siol et al. 2010).

For the estimation of parameters of species divergence in the isolation-with-migration framework (Hey and Nielsen 2004), various approaches have been implemented, including Markov chain Monte Carlo methods such as LAMARC (Kuhner 2006), MIMAR (Becquet and Przeworski 2007), IM, and subsequent developments of the latter (Hey and Nielsen 2004, Hey and Nielsen 2007; Hey 2010; Choi and Hey 2011). A hidden Markov model was introduced by Mailund et al. (2011) to estimate the divergence time and recombination rates along an alignment of two genomes excluding gene flow. More flexible in the underlying demographic model are approaches such as the diffusion approach ∂a∂i (Gutenkunst et al. 2009), the composite-likelihood method of Garrigan (2009), and approximate Bayesian computation (ABC) methods (e.g., Beaumont et al. 2002; Beaumont and Balding 2004; Bazin et al. 2010).

The assumption of an infinite-sites mutation model (ISM) is critical for the computation of the likelihood in many classical and recent population genetic approaches (Kimura 1969; Watterson 1975; Gutenkunst et al. 2009; Chen 2012). Apart from rare exceptions, likelihood computations are only possible under this assumption. According to the ISM, all mutations that have occurred along the sequences since the most recent common ancestor of the sample affect a new site; therefore no single position can mutate twice. However, it is not uncommon to observe three or four nucleotides segregating at a single site in data sets, indicating a clear violation of ISM. A widely used approach is to exclude these sites from further analyses. This procedure may be reasonable if only a few positions show multiple hits. Moreover, not all double hits will be visible in the sequence alignments, and neglecting them biases estimates of the population mutation parameter *θ*. Desai and Plotkin (2008) concluded that if *θ* per site exceeds 0.05, neglecting back mutations and multiple mutations (in the following termed *neglecting finite sites*) will increase the false positive rate in tests for selection.

Multiple mutations can have several effects on parameter estimations. For example, migration rates may be overestimated because independent mutations at the same nucleotide position can be interpreted as a migration event. Likewise, back mutations (reversals) on long branches could cause these branches to appear shorter. This will affect estimates of divergence times and population growth. If a parallel mutation occurs on the branch leading to the outgroup, the ancestral state will be misidentified which can affect the determination of ancestral and derived states.

Mutation rate heterogeneity between sites can compound the problem of multiple hits by increasing the rate of undetected double hits, thereby leading to a mis-estimation of *θ* and other parameters. Rogers and Harpending (1992) showed that based on the shape of the distribution of the number of sequence polymorphisms, it is possible to estimate the timing and extent of population expansion. They applied this approach to study human mitochondrial data but assumed infinite sites. Subsequently, several authors noted that the ISM assumption is not met in the case of mitochondrial data (e.g., Lundstrom et al. 1992; Aris-Brosou and Excoffier 1996; Schneider and Excoffier 1999). Furthermore, Aris-Brosou and Excoffier (1996) observed that mutation rate heterogeneity affects the number of segregating sites in a way similar to a recent population expansion. Using an ISM while neglecting rate heterogeneity can lead to deviations in parameter estimation up to 20% in a simple expansion model and can have a severe effect on the estimation of confidence intervals (Schneider and Excoffier 1999). In *A. thaliana*, models that include variable mutation rates fit better than models without (François et al. 2008).

MCMC-based programs like IM (Hey and Nielsen 2004) or LAMARC (Kuhner 2006) have not only the advantage of using considerably more information from the sequence data than summary statistics-based methods but can also include finite-sites mutation models (FSM) in their estimation. Well-known examples of FSMs from simple to more complex models are Jukes Cantor (JC), Kimura-2-parameter, Felsenstein 81, Hasegawa Kishino Yano (HKY), and the general time reversible (GTR) model (Jukes and Cantor 1969; Kimura 1980; Felsenstein 1981; Hasegawa et al. 1985; Tavaré 1986). A limitation of “full-data” methods, such as IM and LAMARC, is, however, that it is difficult to extend them to population demographic models outside their intended range. Moreover, full-data methods are computationally very demanding, which makes them inappropriate for large NGS data sets.

**Figure 1:**
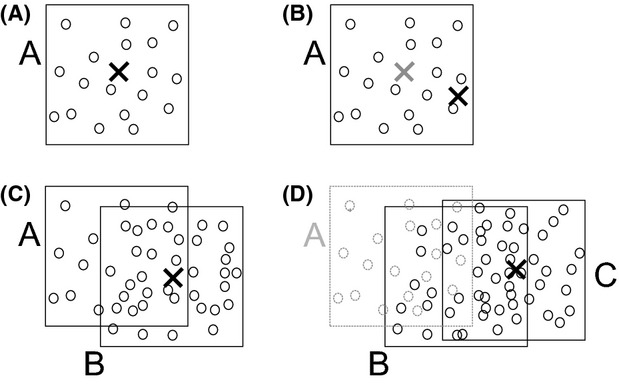
Refined search strategy in Jaatha 2.0. (A) Box A is a cube in the parameter space that is placed around the initial estimate 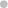 (×). *s*_*main*_ parameter combinations (bullets) are sampled from box A and are used for simulations, which are then (B) used to refine the parameter estimation (black ×). (C) Box B is placed around the refined parameter estimate and new simulations are launched with parameter combinations sampled from box B. These simulations are combined with the previous ones to further refine the parameter estimations. (D) As the current parameter estimation moved out of box A, only simulations according to parameter combinations sampled in box B and in box C are used for the next iteration. Simulation results from box A (gray) are deleted.

In Naduvilezhath et al. (2011), we introduced the composite-likelihood method Jaatha, which estimates demographic parameters of two recently diverged species from polymorphism data. Similar to the ABC approach (e.g., Beaumont et al. 2002; Leuenberger and Wegmann 2010), Jaatha uses simulations for a range of parameter values to assess how the summary statistics (SS) depend on the parameters of the demographic model. Although Jaatha is flexible regarding the demographic model, simulating the entire parameter space a priori is only feasible with a maximum of four model parameters. For more complex demographic scenarios, estimating four parameters is too limiting. Here, we present a new version of Jaatha that has no strict limitation on the number of parameters of a user-defined speciation model. The main modification compared to the previous version is that after an initial coarse search, the program applies an adaptive strategy to launch simulations for regions of the parameter space that are most relevant for the observed data set (Fig. 1).

Using simulated data, we investigate the effects of assuming the ISM when the data are generated under an FSM. We find that assuming an ISM in the presence of FSM can lead to an overestimation of the divergence time and the migration rates and an underestimation of *θ*. Parameter estimations improve considerably when a finite-sites sequence simulator (such as Seq-Gen by Rambaut and Grassly 1997) is included in the method. In this case, Jaatha can provide accurate estimates of FSM parameters such as the mutation rate heterogeneity.

**Figure 2:**
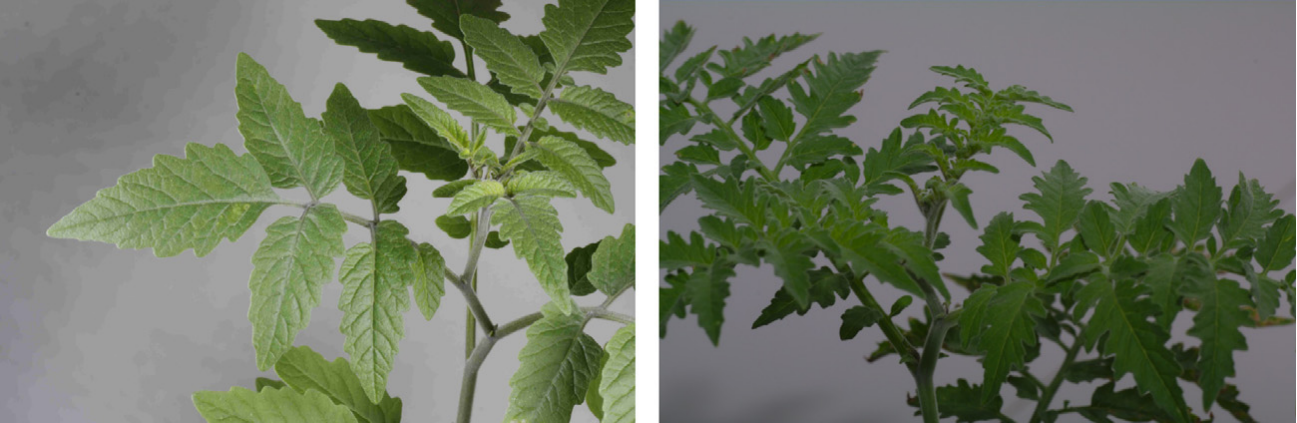
*Solanum peruvianum* and *Solanum chilense*. Leaf morphology of two focal species *S. peruvianum* from LA2744 (Sobroaya, Chile) and *S. chilense* from LA2884 (Ayaviri, Chile).

To demonstrate the improvements in the new version of Jaatha, we reanalyze the example data set used in Naduvilezhath et al. (2011). It consists of DNA alignments from seven genes of the wild tomato species *Solanum chilense* and *S. peruvianum* (Fig. 2). A fraction of the polymorphic sites (7.3% or 70 positions) showed three or four different nucleotides across the sampled sequences including the outgroup sequences, and therefore two or more mutational events must have occurred at these sites. This high number of affected sites suggests that we should account for back mutations and double hits when analyzing the *Solanum* data. Although strong hybridization barriers exist between these species and hybrids have not been observed in the natural habitat (R. Chetelat, personal communication), our previous analysis of the seven genes yielded significant nonzero interspecific migration rates for all models (Naduvilezhath et al. 2011).

In simulations, we show that migration rates can be severely overestimated by assuming the ISM. With the new versions of Jaatha, we were able to explore two alternatives for observing the apparent gene flow between *S. chilense* and *S. peruvianum*: (1) The signature of migration between species could be due a gradual decrease in gene flow after divergence (“Decreasing Migration” model, see below) or (2) The signature of migration is an artifact of neglecting FSM. Using a simulation-based likelihood-ratio test, we show that migration rates are still significantly different from zero if we take multiple hits into account.

Since Jaatha needs relatively few initial simulations, the analysis of large NGS data is possible. As a proof of concept, we apply Jaatha to a genome-wide data set of two southern European *A. thaliana* populations and a Siberian population, which served as an outgroup. Our results suggest that the southern European populations have split long before the last ice age. This is in contrast to the proposal in Sharbel et al. (2000) of a more recent split in these populations during the last glacial maximum in Europe.

## Material and Methods

### Demographic models

**Figure 3:**
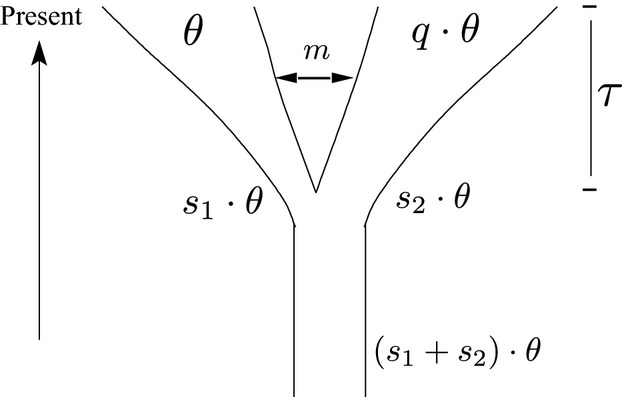
Basic demographic model. In this speciation model, a single ancestral population splits into two populations *P*_1_ and *P*_2_. All size ratios are relative to *N*_1_, *θ* = 4*N*_1_*μ* and *μ* is the mutation rate per generation per locus. *P*_1_ grows exponentially after the split from the size ratio *s*_1_ to its present size and shrinks if *s*_1_>1. *P*_2_ starts immediately after the split with a size ratio of *s*_2_ and grows or shrinks exponentially to reach the present day size ratio of *q*. Besides the size ratios *q*, *s*_1_, and *s*_2_ between the two populations, the model is parameterized by the population mutation rate *θ*, the divergence time *τ*, and the symmetric migration rate *m*. The last three parameters are scaled with 4*N*_1_ following the parameterization in Hudson's ms program (Hudson 2002).

In our basic model (Fig. 3), we consider two populations *P*_1_ and *P*_2_ of sizes *N*_1_ and *N*_2_=*q*×*N*_1_. The populations emerged *τ*×4*N*_1_ generations ago from a joint ancestral population of size (*s*_1_+*s*_2_)×*N*_1_. Both populations can experience a size change: *P*_1_ from size *s*_1_×*N*_1_ to its present day size *N*_1_ and *P*_2_ from size *s*_2_×*N*_1_ to *N*_2_. When *s*_1_=1 (or *s*_2_=*q*), no size change occurs in *P*_1_ (*P*_2_). A symmetric migration rate *m* between the species is assumed, which is scaled with 4*N*_1_, such that on average each generation 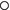 individuals replace inhabitants of the other population. The population mutation parameter is defined as *θ*=4*N*_1_*μ*, where *μ* is the mutation rate per locus per generation. In Section S1.1, we give the command line to simulate population genetic data according to this model for the program ms (Hudson 2002), which is a backward-time coalescent simulator, and specify the parameter ranges that we used. We will now introduce several models that are nested in the basic model. An overview of these models along with the results is given in Table 2, in which parameter values that are fixed in a particular model are shown bold.

The models “Constant” and “Fraction-Growth” contain four parameters: *θ*, divergence time *τ*, present day population size ratio *q*, and migration rate *m*. In both models, *s*_1_=1 is fixed. In the model “Constant”, we fixed *s*_2_=*q*; therefore, population size changes in *P*_2_ after the split are not permitted. In the model “Fraction-Growth”, *s*_2_ is fixed to 0.05 and the population size of *P*_2_ is allowed to change. The following models were fit to the tomato data: In the model “FixedS2”, four *main* parameters are estimated *θ*, *q*, *τ*, and *m*. The parameters *s*_1_ and *s*_2_ are fixed to 1 and 0.3, respectively, implying a size change in *P*_2_ only. The model “NoMig” differs from the model “FixedS2” in that *m* is not estimated but fixed to 0. In the model “SingleGrowMig”, the parameter *s*_2_ is estimated in addition to the ones described for the model “FixedS2”, thus allowing for a size change in *P*_2_. In the model “BothGrowNoMig”, the migration rate *m* is set to 0, and *s*_1_ is included into the parameter space compared to “SingleGrowMig”. In the model “BothGrowMig”, two parameters, *s*_1_ and *s*_2_, are estimated in addition to the four main ones.

**Figure 4:**
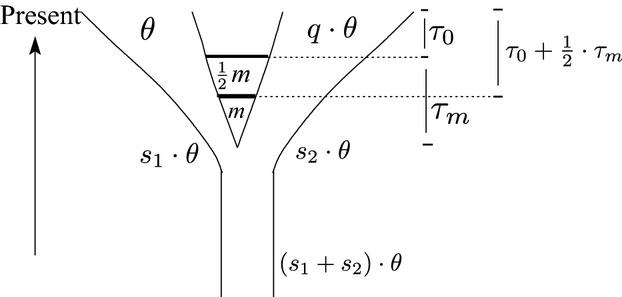
“Decreasing Migration” model. Seven parameters of this model were estimated: population mutation rate *θ*, divergence time *τ*, size ratio between the present day population sizes *q*, starting size of *P*_1_ and *P*_2_ relative to *N*_1_ immediately after the split *s*_1_ and *s*_2_, symmetric migration rate *m* following the split, and two times, *τ*_0_ and *τ*_*m*_. Characterizing the migration behavior from the past to the present, directly after the split during the time span *τ*_*m*_ there was symmetrical gene flow between the two populations at rate *m*. At time 0.5×*τ*_*m*_, migration decreases to 0.5×*m*. During the most recent time span, *τ*_0_, there was no migration between the populations. All population sizes are relative to that of *P*_1_.

As an example of a model with seven parameters, we assessed the accuracy of the parameter estimation in the “Decreasing Migration” model (Fig. 4). The model “Decreasing Migration” is different from the basic model in that the migration rate *m* between both populations decreases in two steps from *m* to zero. The time span following the split of both populations in which gene flow occurs is denoted *τ*_*m*_. At time 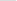 before present, the migration rate is set to half of its value. *τ*_0_ denotes the time point at which gene flow has decreased to zero. The ms command line is given in Section S1.2.

### New version of Jaatha

The aim of Jaatha is to estimate a set of *n* parameters of a speciation model of two species *P*_1_ and *P*_2_ from a data set *D* of homologous DNA sequences sampled from *y*_1_ gametes from *P*_1_ and *y*_2_ from *P*_2_. We summarize the data set *D* with a set of SS from the two-dimensional joint site frequency spectrum (JSFS) *J*. The JSFS counts the number of single-nucleotide polymorphisms (SNPs) in *D* for which the derived allele occurs in each population, for example *J*[*a*,*b*]=*j*_*ab*_=5 which means that there are 5 positions in *D* at which the derived allele is found in exactly *a* individuals of *P*_1_ and in *b* individuals of *P*_2_. On the JSFS, we define a set of SS 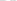, where 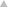 and 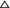 is a partition of *A*={0,…,*y*_1_}×{0,…,*y*_2_}∖{(0,0),(*y*_1_,*y*_2_)}. In the following, we use *n*_*SS*_=23. For the partition, we chose the high- and low-frequency polymorphisms to be binned in a similar fashion because polymorphism at sites with mutations on the branch leading to the outgroup affect those in particular. An example of the *A*_*i*_, descriptions with *y*_1_=*y*_2_=10 can be found in Figure S3. Since Jaatha draws parameter values uniformly from the log-scaled parameter space, the following parameter values are specified on log scale, and the same holds for the set of true parameter values **p**=(*p*_1_,…,*p*_*n*_). Jaatha is a composite-likelihood method, which means that the likelihood is approximated by assuming unlinked SNPs (Kim and Stephan 2000; Hudson 2001; McVean et al. 2002). Hence, our SS are Poisson distributed. Thus, we can compute the composite likelihood for a parameter combination 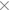 by


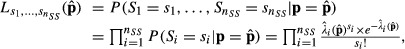
(1)

where 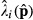 is our estimate for the expected value 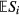. Here, *L* is a *composite* likelihood because dependencies between the SS are neglected. For the calculation of 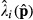, we first simulate data sets in a specific parameter space 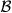 for which we calculate the SS 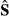. We then fit to each of the 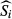 a Poisson generalized linear model (GLM) with log link using the *glm()* function in R (R Development Core Team 2009). This GLM describes how the expectation values of 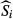 depends on the log-scaled parameters 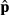 in 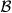. The parameter values 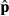 in 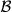 that maximize the approximate Poisson probability 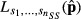 (eq. 1) of **S** are determined with the *optim()* function in R using the optimization procedure of Byrd et al. (1995).

The new version of Jaatha consists of an initial and a refined search: Initially, we fit GLMs to data simulated for large regions of the parameter space to find promising starting points for the subsequent refined optimization procedure. In the following paragraphs, we give a more detailed explanation of the two phases and the settings that can be specified by the user.

1. Initial Search: Finding good starting positions. First, we divide the parameter space into equally sized blocks by dividing each parameter range 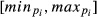 into *k* intervals such that we obtain *k*^*n*^ blocks with 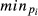 and 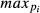 being the minimum and maximum of the parameter range for parameter *p*_*i*_ and *i* ∈ [1,*n*]. Within each block using Hudson's ms (Hudson 2002), we simulate *s*_*ini*_ data sets of *n*_*loc*_ loci with, on the log scale uniformly (in the following simply uniformly drawn) drawn parameter values. For all data sets, we calculate SS and fit a GLM to each SS. With these GLMs within each block, we can find the parameter combination that maximizes the score of the observed SS. Each of the *k*^*n*^ blocks provides a single best parameter combination. Out of this list, *n*_*RP*_ starting positions (default *n*_*RP*_=10) points with the highest score 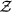

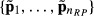
 are selected for the in-depth-search.
2. Refined Search: Finding *n*_*RP*_ best point estimates. For *b* ∈ {1,…,*n*_*RP*_} do:
  - Assembling a list 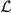 of best parameter estimates starting from 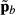 (Fig. 1): Around 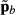, we perform a *Jaatha step* to obtain 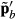: First, we define a block 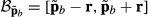, where *r*_*i*_=*r* for all *i* ∈ 1,…,*n*. Within this block 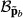, we simulate *s*_*main*_ data sets of *n*_*loc*_ loci with uniformly chosen parameters from within this block (corner points in addition), calculate SS, fit GLMs as described above, and estimate a new optimal parameter combination 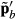. We then run a Jaatha step for a parameter range around 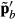 to find 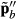. For the GLM fitting to find 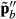, we only reuse simulations of previous blocks if 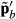 falls within the block, otherwise the simulations are deleted from memory (Fig. 1D). Especially for the FSM runs, this is necessary to reduce the amount of memory usage. This procedure is iterated until the score of the new parameter combination has not changed by more than *ε* in any of the last *t*_*stop*_ steps. The maximum number of steps can be specified as another stopping criterion (*t*_*max*_) which was necessary in particular when *ε* was small such that the score did not seem to converge. Throughout this phase, we keep a list (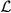) of *n*_*B*_ parameter combinations with the highest scores. There is an option to weight simulations of blocks with *w*^*i*^, where *w* ∈ [0,1] and *i* states how many iterations ago the simulation was performed.
  - Evaluation of the parameter estimates in 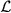: After phase 2 (a) has finished, the parameter combinations stored in 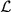 will be used to perform *s*_*final*_ independent simulations for each of them to calculate the composite-likelihood of each parameter combination (using eq. 1) with
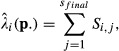
    where 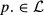 and *S*_*i*,*j*_ is the *i*-th SS of the *j*-th simulation. The parameter combination with the highest likelihood will then be reported as the result for *b*.

Since we start the detailed search for each of the *n*_*RP*_ refine points, Jaatha will report *n*_*RP*_ parameter combinations in total. The Jaatha results in the following always represent the parameter combination with the overall highest likelihood.

Another option that can be set by the user is *ext*_*θ*_, which specifies whether *θ* is excluded from the parameter range from which the random values are chosen for the simulations. If this option is set, *θ* is fixed to the value of 5 for the simulations, which reduces the dimensions of block 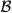 by one, while the other parameters are calculated as described above. *θ* is then estimated separately of the other parameters as in Naduvilezhath et al. (2011). Note that this approach is based on an ISM heuristic.

An implementation of the algorithm can be downloaded as an R package (R Development Core Team 2009) from http://evol.bio.lmu.de/_statgen/software/jaatha/ or from CRAN (http://cran.rproject.org/web/packages/jaatha/index.html). An intensive simulation study to optimize Jaatha settings was carried out. The description and results are included in the Section S2.

### Example data sets

#### Solanum data set

*S. chilense* and *S. peruvianum* are diploid perennial plants that inhabit the Western Coast of South America. All *S. chilense* and *S. peruvianum* analyses were performed on the seven loci of average gap-free length of 1264 bp with a total of 954 SNPs from on average 44 alleles per locus of *S. chilense* and 43 alleles per locus of *S. peruvianum* (Arunyawat et al. 2007; Städler et al. 2008). The outgroup sequence for all genes was *S. ochranthum*, which diverged from the ancestor ≍5.8 to 13.6 million years ago (L. Rose, unpubl. data). In all FSM-estimations with the *Solanum* loci, we use the “*Solanum* configuration”. We define the “*Solanum* configuration” as follows: The nucleotide frequencies *p*(.) are set to those observed in the *S. chilense* and *S. peruvianum* data set: 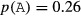, 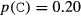, 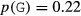, and 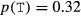. We used *ti*/*tv*=2 for the simulations. (A value of *ti*/*tv*=1.6 is observed when the *S. chilense* and *S. peruvianum* are compared to the outgroup sequence.) The divergence time factor *T* (explained in the following) is set to 2 and the sample sizes to 44 and 43. When the Γ-shape parameter *α* was not estimated by Jaatha, it was set to a value of 0.7, which is the average value suggested by Modeltest 3.7 (range from 0.46 to 1.09 in analyses of the *Solanum* genes; Posada and Crandall 1998). The transition–transversion ratio *ti*/*tv* is defined as the ratio of expected numbers of transitions and transversions such that, for example, *ti*/*tv*=0.5 for the Jukes Cantor model (Jukes and Cantor 1969). For both the simulation studies and the simulations for the analyses of the tomato data, the ancestral states of each site were inferred from outgroup sequences simulated based on a divergence time of *T*×*τ* from the present. The shape parameter *α* of the Γ-distribution models how the mutation rate varies across the sites (the scale parameter *β* of the Γ-distribution is fixed to 1/*α*). Small values of *α* correspond to greater rate heterogeneity between sites (for the chosen parameter range for *α* see Section S1.3).

To test whether migration rate was significantly different from zero, we followed a likelihood ratio testing approach with null model having no gene flow (as mentioned by Hey 2006). For this, we calculated the composite log-likelihood ratio ℓLR, that is 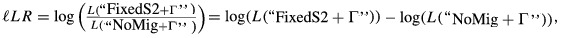, where *L* is the composite likelihood of the specified model. This yielded a ℓLR of ≍ 14 for the *Solanum* data. Since we used *composite* likelihoods, we could not apply a *χ*^2^ approximation to calculate *P*-values but instead used a simulation procedure (Naduvilezhath et al. 2011). We tested how often we would observe a ℓLR as high or higher if the data were simulated under the assumption of no gene flow. We simulated 200 sequence files with the best “NoMig+Γ” parameter estimates for the *Solanum* loci under the “*Solanum* configuration”, a recombination rate per locus of 25, and sequence length of 1250 bp. These data sets were then analyzed in the same way as the *Solanum* data: We used the “FixedS2+Γ” and “NoMig+Γ” model to calculate the ℓLR of the best parameter estimates. The Jaatha settings for these analyses were the same as for the *Solanum* data (J7 for the “FixedS2+Γ” model and the “NoMig+Γ” model) but with *n*_*RP*_=10 for the “FixedS2+Γ” model. In Naduvilezhath et al. (2011) we also performed a likelihood-ratio test comparing two FSMs, which showed significant evidence for gene flow. The difference of the analysis conducted here to the previously used FSM model was that *α* and *ti*/*tv* were not fixed but estimated from the *Solanum* data as well.

For the best fitting model “FixedS2+Γ”, we constructed bias-corrected bootstrap confidence intervals as described by Efron and Tibshirani (1993). We simulated 100 bootstrap data sets of 7 loci with the recombination rate *ρ*=5 per locus per 4*N*_1_ generations, which was the lowest value of *ρ* estimated for the tomato loci (Naduvilezhath et al. 2011, Suppl.). Increasing *ρ* would make the confidence intervals narrower because the data will be more unlinked and thus decrease the variances of the SS. Therefore, our confidence intervals are conservative. The other simulation details were set as in the composite-likelihood ratio test. In Naduvilezhath et al. (2011) we demonstrated with a seven loci meta-bootstrap analysis that bootstrap confidence intervals have an accurate coverage probability. To reduce run time, we fixed *α* to 2.5, which is the *Solanum* estimate under this model.

#### Arabidopsis thaliana data set

As a NGS application, we analyzed *A. thaliana* genome sequences from 12 individuals from Italy, 12 individuals from Spain, and 5 individuals from Novosibirsk presented in Cao et al. (2011). We used loci for which homologous sequences were available from all three populations (for accession numbers see Table 1). After filtering out missing, ambiguous, and nonpolymorphic positions, we obtained more than 1.1 million SNPs. We divided them into three groups based on the level of selective pressure that we assume they experience. The first group (FS) consisted of **f**irst and **s**econd codon positions and UTRs, whereas the second one (Th) consisted of **th**ird codon positions. The last category (NC) contained **n**on**c**oding positions (introns and intergenic regions). If a SNP could be assigned to more than one group – for example, because of overlapping genes – we assigned it to the more conserved group. We applied Jaatha to the complete data set, as well as to each group separately, using an HKY model with estimated base frequencies and *ti*/*tv* ratio (Jaatha setting J17) as well as an ISM (Jaatha setting J18). We assumed a demographic model with a split between the two southern European populations and subsequent migration, a constant mutation rate, equal and constant population size of each of the contemporary populations, and the ancestral one. The Siberian population was used as an outgroup.

**Table 1:** Accession numbers of examined *Arabidopsis thaliana* samples

Sample	Accession Numbers
Italy	Agu-1, Cdm-0, Don-0, Fei-0, ICE49, ICE50, Leo-1, Mer-6, Ped-0, Pra-6, Qui-0, Vic-0
Iberia	ICE91, ICE92, ICE93, ICE97, ICE98, ICE102, ICE104, ICE106, ICE107, ICE111, ICE112, ICE120
Outgroup	ICE127, ICE130, ICE134, ICE138

Cao et al. (2011) for further reference.

## Results

### Relationship between the number of sampled loci and the ability to estimate population genetic parameters

We investigated the effect of the number of loci sampled on the ability to accurately recover estimates of the population genetic parameters under the “Decreasing Migration” model. Data were simulated for seven loci matching the “*Solanum* configuration” (see above) with an HKY+Γ model and for 200 (ISM-) loci (for details of parameter ranges and ms command see Section S1.2). On these data sets we applied Jaatha assuming an ISM, therefore neglecting the fact that the data were generated under an FSM. The simulated data sets were analyzed with Jaatha setting J1 (Table S1 with *n*_*RP*_=16).

**Figure 5:**
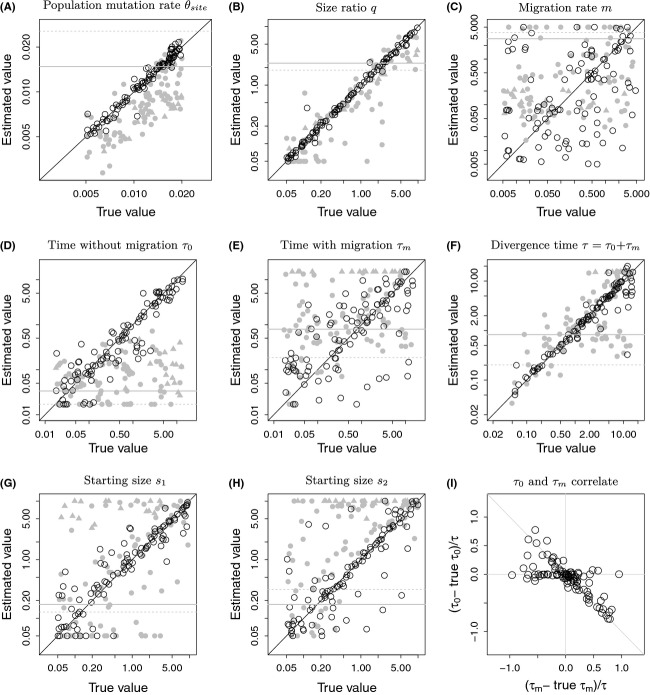
Parameter estimation under the “Decreasing Migration” model with 7 loci is imprecise but improves with additional loci. Results with simulated data (7 (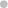) and 200 (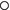) loci) and tomato loci (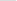 with FSM, 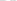 with ISM) with the “Decreasing Migration” model with seven parameters. In the case of 7 loci, when *τ*_*m*_ is estimated to be >15 (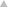), parameter estimates are particularly imprecise. (D) Further, *τ*_0_ is never estimated to be greater than ≍0.7, a behavior that does not occur when 200 loci are used. (F) The divergence time *τ* is calculated by *τ*_0_+*τ*_*m*_ and is more precisely estimated than *τ*_0_ and *τ*_*m*_ separately. (I) In the 200 loci case, if *τ*_0_ is not calculated correctly the estimates of *τ*_0_ and *τ*_*m*_ correlate negatively such that their sum equals the divergence time *τ* again.

In Figure 5, we show that Jaatha can estimate seven parameters accurately if enough loci are available. In the case of seven loci, great uncertainty is associated with all parameter estimates, especially large values of *τ*_0_ were dramatically underestimated (always ≤0.7, Fig. 5D). The corresponding value for *τ*_*m*_ is typically overestimated and reaches the upper limit of the parameter range of *τ*_*m*_, such that the estimate of the divergence time *τ*_0_+*τ*_*m*_ is quite accurate, even for seven loci (Fig. 5F). For the case of 200 loci, the more recent time *τ*_0_ can be estimated more accurately than *τ*_*m*_. We observed no obvious connection to migration rate. The estimation errors of *τ*_0_ and *τ*_*m*_ were negatively correlated (Fig. 5I). When sufficiently many loci are available, the parameters *θ*, *q*, and *τ* can be estimated quite confidently; with slightly more fluctuation in accuracy, *τ*_0_ and the starting sizes after the split of both populations, *s*_1_ and *s*_2_ can also be estimated. The influence of different settings on the accuracy and run time of Jaatha has been extensively studied under a simple demographic model. The analyses and results are presented in Section S2.

### Violations of the infinite-sites model cause overestimation of divergence time and migration rates

**Figure 6:**
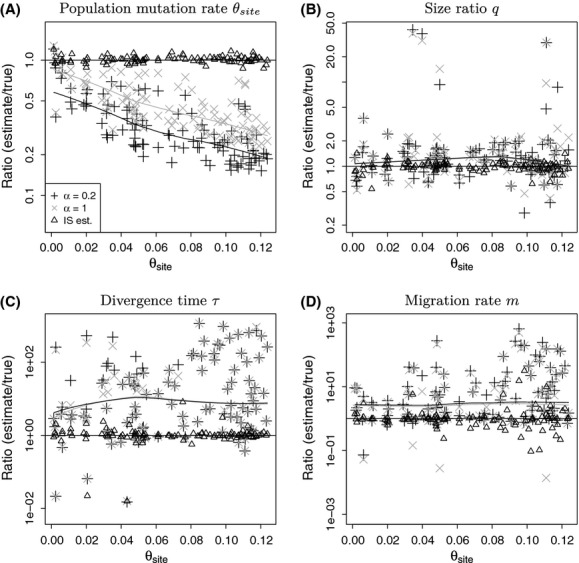
The effect of neglecting finite sites on parameter estimation under the “Fraction-Growth” model. The ratio of estimated and true values of *θ*, *q*, *τ*, and *m* plotted against true *θ* values under infinite-sites assumptions and the "Fraction-Growth“ model. Shown are the data sets simulated with the most extreme *α* values (*α*=0.2 and 1), *ti*/*tv*=2, and *T*=3. As a comparison, estimates for infinite-sites data sets (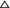) are included. The lines plotted are polynomial regression lines fitted to the ratios (with *lowess* function of R). The greatest influence of neglecting finite sites was observed in the estimates of *τ* and *m* (notice different scaling of Y-axes).

We conducted the following simulation study to assess the quality of the estimations and to determine which parameters were most affected if we neglect back mutations and double mutations and analyze the data under infinite-sites (IS) assumptions: Using ms (Hudson 2002), we constructed genealogies based on 100 loci under the “Constant” and “Fraction-Growth” model (see Section S1.1). To simulate the evolution of nucleotide sequences for these genes, we used Seq-Gen (Rambaut and Grassly 1997) under a HKY + Γ model with the “*Solanum* configuration”, with transition–transversion ratio *ti*/*tv* and the outgroup divergence time factor *T* variable (Hasegawa et al. 1985). For the simulation study, we tested three values of *ti*/*tv*: 1, 2, and 5. Five values for the Γ-shape parameter *α* were chosen: 0.2, 0.3, 0.5, 0.7, and 1 (The estimated *α* found in the literature for genes from vascular plants ranges between 0.18 and 0.78 and for *ti*/*tv* between 2.6 and 5.3 (Soltis et al. 2002).). For explanations of *ti*/*tv*, *T*, and *α* see above. Hence in total, we simulated data under 15 HKY models (3 values of *ti*/*tv*, 5 of *α*). Additionally with each model, three different values for *T* were chosen (1.5, 3, and 6) to see if they had an impact on the results. To account for possible variation in the sequences for each genealogy, five data sets were simulated (repetitions). The value of *θ* for the simulated data sets ranged from 1.25 to 125 per locus (0.001–0.1 per site; for other parameter ranges see Section S1.1). Jaatha defines a nucleotide to be derived when it is different from the outgroup sequence. In total, we analyzed 27,000 data sets with four methods of a previous verison Jaatha 0.2 (described in Tellier et al. 2011) under the assumption of an ISM to estimate four parameters. Thus, we carried out 1.08×10^5^ Jaatha runs (all combinations of 15 HKY models, 3 values of *T*, 2 demographic models, 100 data sets, 3 repetitions, and 4 Jaatha methods). This large number of runs was only feasible because we applied Jaatha 0.2, which allows us to reuse the simulation results of the first phase. In Figures 6, S5, and S6 the average over the repetitions are plotted.

As values of the true population mutation rate *θ* increased, *θ* was increasingly underestimated (*e.g.,* Fig. 6A). The estimation accuracy for the size ratio *q* was the least sensitive to increasing values of *θ* (Fig. 6B), although for high true values of *θ*, *q* was overestimated by up to 50% in the “Fraction-Growth” model. The parameter estimation of divergence time *τ* and the migration rate *m* were affected the greatest as true *θ* (hence the rate of multiple hits) increased. Both parameters were overestimated by up to three orders of magnitude (Figs. 6C and D). As *θ* increased, all estimations had higher variances. The misestimation of *τ* and *θ* was particularly severe for low values of *α*, the mutation rate heterogeneity parameter.

Neglecting finite sites affected the misestimations of the parameters in the two demographic models “Fraction-Growth” and “Constant” differently (cp. Figs. 6 and S5). The overestimation of the divergence times was greater under the “Fraction-Growth” model than under the “Constant” model; however, the ability to properly estimate the migration rate was not much affected. The size ratio estimate *q* showed a greater number of extreme outliers in the demographic model with population growth than in the one without (cp. Figs. 6B and S5B). The transition–transversion ratio *ti*/*tv* (Fig. S6) and the divergence time factor *T* had no obvious influence on the estimates (data not shown).

In general, when *θ* was above a value of 10 per locus (≍0.01 per site), the estimates worsened compared to the estimates of data sets simulated and evaluated under the correct model. For data sets with *θ* estimates above this critical value, we propose that a finite-sites simulator should be used for the simulation procedure. For data sets with lower mutation rates, bias corrections based on the observed regression lines might be a possibility to obtain results faster, but will be imprecise.

### Jaatha estimates mutation rate heterogeneity accurately

**Figure 7:**
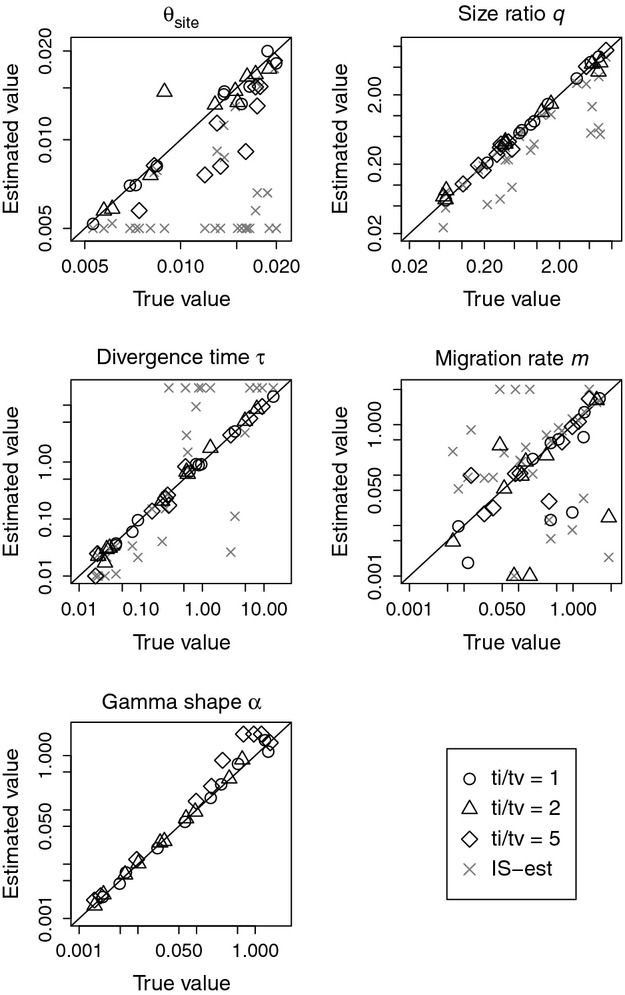
Estimation of Γ shape parameter jointly with demographic parameters. Here, we estimated four demographic parameters and the Γ shape parameter on 30 simulated data sets containing 100 loci, each with 30 summary statistics. For the estimation, the transition–transversion ratios (*ti*/*tv*=1,2,5) were fixed to the true value. Shown are also the estimates of the infinite sites (IS) runs with Jaatha on the same data sets (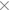). A clear drop in precision of the estimates of all four parameters is observed if an IS model is chosen instead of an finite-sites model.

To estimate parameters under an FSM with Jaatha, we simulated data with ms (Hudson 2002) in conjunction with Seq-Gen (Rambaut and Grassly 1997) in the initial and refined search phase. To determine how well Jaatha is able to jointly estimate FSM parameters, in particular the Γ shape parameter *α*, in combination with demographic parameters, we simulated ten data sets each for three different values of the transition–transversion ratio *ti*/*tv* (1, 2, 5). The estimation of *α* in combination with the other parameters was accurate (Fig. 7; for details see Section S1.3). Parameter estimation improved greatly compared to the results obtained by applying an ISM in Jaatha on the same data sets. For high values of *ti*/*tv*, the estimation of *θ* and *α* became less accurate, even if it was based on FSM. For the *Solanum* loci, we estimated *ti*/*tv*≍1.6 based on the observed number of transitions and transversions relative to the outgroup. In this *ti*/*tv*-range, parameter estimation is robust if enough loci are available. (The ISM runs were run with two different Jaatha settings that yielded similar results, therefore only the results with one of them, J5 (see Table S1), are shown.).

### Application example I: Speciation in *Solanum*

Our simulation results in Figure 5 indicate that estimating the seven parameters of the “Decreasing Migration model” from only seven genes is quite imprecise for all parameters. Therefore, we will not discuss the *Solanum* results for this model in much detail, but mention that the migration rate between species was estimated to be extremely high and the time without migration *τ*_0_ to be extremely recent (Table 2). According to our analysis of the wild tomato species with models in which fewer than seven parameters were estimated, the ancestral population size ratio (*s*_1_) of *S. chilense* was below one (0.17), indicating an expansion in this species following speciation. Previous studies have not uncovered a signal of an expansion in this species, but rather in *S. peruvianum* (Städler et al. 2008). Therefore, we attempted to determine how strong the signal for expansion was in *S. chilense* (and whether our data set contained sufficient information to distinguish between alternative scenarios). We analyzed the *Solanum* data with three additional models: (1) “SingleGrowMig” in which only *S. peruvianum* experienced an expansion and gene flow was present between species, (2) “BothGrowNoMig” with an expansion in both species but with no gene flow between species, and (3) “BothGrowMig” with an expansion in both species and gene flow. Although the latter two models contained more parameters, the “SingleGrowMig” model fit the wild tomato data best. Hence, the indication of growth in *S. chilense* is not supported. The two best fitting models in which FSM is applied were the “FixedS2+Γ” and the “SingleGrowMig” model. For the estimates of the “FixedS2+Γ” model with *α* fixed to 2.5, the 95% bias-corrected bootstrap confidence intervals are given in Table 3.

**Table 2:** Estimated parameter values and log-likelihoods based on sequence data from *Solanum chilense* and *Solanum peruvianum*

Model	*θ*_*site*_	*q*	*m*	*τ*	*s*_1_	*s*_2_	*α*	# Parameters	log-Likelihood	Settings
NoMig	0.012	10	**0**	0.17	**1**	**0.3**	**0.7**	3	−91.6	J8
NoMig+Γ	0.012	10	**0**	0.21	**1**	**0.3**	0.69	4	−83.2	J7
FixedS2	0.012	10	0.04	0.22	**1**	**0.3**	**0.7**	4	−81.5	J9
(IS) FixedS2	0.010	4.65	0.59	0.39	**1**	**0.3**	–	4	−∞	J10
FixedS2+Γ	0.010	6.05	0.36	0.32	**1**	**0.3**	2.5	5	−69.2	J7
SingleGrowMig	0.011	5.67	0.46	0.39	**1**	0.21	**0.7**	5	−69.1	J11
SingleGrowMig+Γ	0.010	6.75	0.41	0.29	**1**	0.24	2.5	6	−72.8	J11
BothGrowNoMig	0.012	5.13	**0**	0.13	0.42	0.58	**0.7**	5	−94.6	J11
BothGrowNoMig+Γ	0.014	3.73	**0**	0.09	0.14	0.19	0.19	6	−294.5	J11
BothGrowMig	0.011	4.47	0.75	0.60	0.62	0.03	**0.7**	6	−87.1	J12
BothGrowMig+Γ	0.016	2.41	0.96	0.24	0.10	0.18	1.11	7	−96.8	J13
DecMig	0.012	2.36	2.79	0.83*	0.17	0.17	**0.7**	7	−87.1	J9
(IS) DecMig	0.020	1.80	3.84	0.20*	0.13	0.31	–	7	−∞	J1

Model	*τ*_0_	*τ*_*m*_
DecMig	0.03	0.79
(IS) DecMig	0.017	0.19

*θ*_*site*_, *m*, and *τ*_(.)_ are scaled by 4*N*_1_, where *N*_1_ is the effective population size of *S. chilense*. Values in bold were fixed for the estimation. In the “+Γ” models, *α* was estimated additionally. The log-likelihoods of the ISM estimates are set to −∞ because the tomato data does not conform to this assumption. The estimates of *τ*_0_ and *τ*_*m*_ are listed in the lower table. See Tables A 1, A 2, and A 3 for Jaatha settings, additional results with alternative settings, and run times.

* This value was calculated after the run with *τ*_0_+*τ*_*m*_.

**Table 3:** 95% confidence intervals for best wild tomato estimates

Model	*θ*_*site*_	*q*	*m*	*τ*	*s*_2_	*α*	# parameters	Settings
FixedS2+Γ	0.010	6.05	0.36	0.32	**0.3**	2.5	5	J7
lower CI boundary	0.008	3.52	0.11	0.14	**0.3**	**2.5**	4	J7
upper CI boundary	0.013	10.51	1.83	0.63	**0.3**	**2.5**	4	J7

The estimates and the 95% bias corrected confidence intervals (CI) for the ”FixedS2+Γ“ estimates are given. For the estimations *α* was fixed to 2.5. Values in bold were fixed for the estimation.

The composite log-likelihood of the “FixedS2+Γ” was by ℓLR=13.95 higher than that “NoMig+Γ”, the corresponding model without gene flow. To access the significance of this evidence of gene flow, we simulated 200 data sets with the “NoMig+Γ” model and analyzed them with both the “FixedS2+Γ” and the “NoMig+Γ” model. Only one of the 200 data sets led to Jaatha results preferring the model with gene flow with equal or higher ℓLR than the 13.95 observed in the tomato data (*P*-value <0.01, range of ℓLR: [−7.22,14.77]). Thus, even when mutation rate heterogeneity is allowed, we still detect significant evidence for gene flow between the two species.

### Application example II: Speciation in *A. thaliana*

When applying Jaatha to a large genome-wide data set from *A. thaliana*, we obtained very recent split times for the population divergence between Spain and Italy and a high rate of gene flow between populations (Table 4 with FSM and Table S4 with ISM). The estimates of these two parameters were nearly the same when we used only the SNPs of any of the classes FS, TR, or NC. Using an ISM for parameter estimation led to slight changes in the estimates. The high migration rates of *M*>3, that is, more than 1.5 individuals per generation makes the genealogies of single loci difficult to distinguish from the standard coalescent of a panmictic population (Gillespie 2004). To test whether the separation between the population is significant, we applied Jaatha with the same population split model to 100 data sets simulated under the assumption that the southern European populations are panmictic. The split times estimated for the panmictic simulated populations were always shorter than the split time estimated from *A. thaliana* data. Thus, the spatial structuring of the Italian and Spanish samples is significant (*P*<0.01).

**Table 4:** Parameter estimates for *Arabidopsis thaliana* using FSM

	*τ*	*m*	*α*	*θ*_*site*_
Complete data set	0.16	3.45	2.87	3.54×10^−3^
FS only	0.12	2.81	4.83	2.73×10^−3^
Th only	0.19	3.31	1.53	3.70×10^−3^
NC only	0.18	3.33	2.26	4.31×10^−3^

Jaatha's estimates using the HKY model for the mutation rate *θ*, time *τ* of the split of both demes, the subsequent migration rate *m* between populations, and the rate heterogeneity parameter *α*. The parameter *τ* is scaled in 2*N*_*e*_ generations, *m* is twice the number of immigrants to each deme per generation, and *θ* is 2*N*_*e*_ times the mutation rate per base.

## Discussion

In this study, we introduced a new version of the composite-likelihood method Jaatha, which estimates demographic parameters of a given model from SNP data. Conducting a simulation study we demonstrated that Jaatha – when applied to sufficiently many loci – gives accurate results under both finite-sites (FSM) and infinite-sites (ISM) models. Jaatha 2.0 is considerably faster than the previous version (couple of hours vs. several days in the ISM case), such that estimations with a finite-site sequence evolution simulator become feasible.

Many population genetic analyses are based on the ISM assumption (e.g., Chen 2012, approaches using diffusion approximations like as Gutenkunst et al. 2009, or ABC methods based on ms Hudson 2002). With increasing values of *θ*, there is a higher probability for back and multiple mutations to occur, some of which will not be observed. MCMC approaches as those implemented in LAMARC (Kuhner 2006) or IM (Hey and Nielsen 2007) do apply finite-sites models (FSM). In currently available software implementations, these methods can, however, take several weeks or months to converge. Moreover, they are restricted to certain types of population genetic models and difficult to extend.

We considered the biologically more realistic finite-sites scenario and investigated the effects of ISM violations on demographic parameter estimations. We show that the divergence time and migration rates are overestimated even for moderate values of *θ*. While Schneider and Excoffier (1999) showed that departures from an ISM could account for a misestimation of the one-population expansion time of 10% to 20%, we observe deviations of divergence time estimates in the two-population scenario of more than two orders of magnitude when *θ* per site exceeds 0.01. If the demographic history includes population expansion, the misestimation is even greater. Thus, failure to account for back and multiple mutations is particularly severe in populations with high-effective population sizes (as it is common in bacteria or plants; reviewed in Charlesworth 2009; Siol et al. 2010) and/or with high mutation rates (high *θ* values). FSM could mimic migration if a mutation occurs in one population and creates a pattern like the one in an individual of the other population. If these two independent mutations are misinterpreted as a single-mutational event, migration may be evoked to explain the presence of the shared polymorphism. This will inflate the estimation of migration.

Multiple mutations create challenges for parameter estimation whether they arise within the genealogy of the population sample or along the lineage leading to the outgroup. A solution to tackling the latter issue was presented in Hernandez et al. (2007). In Jaatha, multiple hits are allowed across the entire genealogy, both within the population sample and along the outgroup lineage; therefore, this distinction based on where the independent mutations occur is not treated explicitly, but is still incorporated into the model. By incorporating FSM into Jaatha, we can control for the problems caused by inappropriately assuming the ISM. In Jaatha, we are able to estimate mutation rate heterogeneity *α* under several simulation scenarios provided enough loci are available. Simulating the demography using ms (Hudson 2002) and subjecting the simulated sequences to a FS sequence generator such as Seq-Gen provided satisfactory results, especially when *θ* is included in the optimization range (Figs. 7 and S2). To decrease the run time of the FSM applications with *α* estimation, there are several possibilities. For example, we have not yet investigated the option of categorizing the Γ shape (-g option in Seq-Gen) as it is commonly done in phylogenetics (Yang 1996). Alternatives to Seq-Gen which are capable of discriminating between coding and noncoding positions are indelSeqGen2.0 (Strope et al. 2009) or SFS_CODE (Hernandez 2008). The latter might be a good alternative because in addition to incorporating FSM into complex demographies, it is also able to apply a distribution of selective effects on newly arising mutations, which will be our next step. Siol et al. (2010) noted that the JSFS might be especially powerful to detect selection.

In a demographic model with two parameters *θ* and divergence times *τ*, large *τ* values (*τ*≥15) were poorly estimated. Since Jaatha is based on a coalescent simulator (ms, Hudson 2002), if the divergence time is larger than the average time that the two populations need to find their common ancestor, Jaatha reaches its limitation (Fig. S4). If gene flow is included into the model, greater divergence times could be resolved. The current version of Jaatha is implemented for complex speciation models of two populations. It is straight forward to apply Jaatha to demographic models of more than two related populations or species, but further investigations for the choice of SS will be needed to obtain good performance in these cases. For FSM, the choice of SS deserves further consideration because reducing the number of SS would save computational time during the run (*e.g.,* with boosting, Lin et al. 2011, or partial least squares (PLS) method, Wegmann et al. 2009; Boulesteix and Strimmer 2007). In Section S3, we describe additional SS for FSMs but there is still room for improvement, especially for high transition and transversion ratios.

Jaatha was applied to the South American wild tomatoes *S. chilense* and *S. peruvianum*. Compared to our earlier estimates when the finite-sites model was not used, our estimates for migration are smaller, but still significantly different from zero. Sousa et al. (2012) showed in a simulation study under an ABC framework that it is possible to distinguish between models with and without migration even with as few as 5 loci. When more loci are available, the accuracy of the parameter estimates increases. In light of the results of our LRT and of Sousa et al., we find evidence for speciation in the presence of gene flow between *S. chilense* and *S. peruvianum*, as has been suggested previously (Städler et al. 2008). However, to answer the question whether gene flow decreased gradually or not (as modeled in the “Decreasing Migration” model), more sequence data is required. With simulated data sets of 200 loci, we show that this is computationally tractable. The size ratio estimate (6.05) is slightly larger and the divergence time (0.32×4*N*_1_, where *N*_1_ is the effective population size of *S. chilense*) between the two wild tomato species is more recent compared to previous estimates. Depending on the generation time (one or seven years), and a per site mutation rate of 5.1×10^−9^ (Roselius et al. 2005), divergence time of the two species is either 0.7 million years (My) or 4.6 My. Our analyses suggest that the population structure of *S. chilense* has not changed size since the split (cp. likelihood of model *SingleGrowMig* of 69.1 and of *BothGrowMig* of 87.1). Interestingly, in the region of the Central Andes where both species cooccur, the Andes underwent a drastic elevation (one third of the present height of the Andes) in the late Tertiary (10 My ago, Jenks 1975). Around 3–5 My ago, a cooling of the temperatures occurred, leading to the formation of the youngest habitat of the Andes and a unique environment for species radiation (*e.g.,* in lupines Smith and Cleef 1988; Hughes and Eastwood 2006; Graham 2009). The timing of the cooling coincides with our divergence estimates of the two species. Therefore, environmental changes in the habitat may have allowed for range expansion of the ancestral species and led to the formation of these two distinct present day taxa.

As a proof of concept, we have applied Jaatha to NGS data from a Siberian and two southern European *A. thaliana* populations. Our estimates for the split time between the Spanish and the Italian populations are very short on a population genetic time scale and the estimated migration rates are very high. With a data set of just a few loci, it would not be possible to distinguish such a scenario from panmixia; however as demonstrated here, the availability of whole-genome data sets makes such distinctions possible. This illustrates the power of such large data sets to understand and extract recent demographic history from genetic information.

According to our results, the split of the Spanish and Italian populations was very recent on a population genetic time scale, but still well before the height of the last glaciation, which was 18−20,000 years ago (Taberlet et al. 1998). If we use experimentally measured rates of about 7×10^−9^ mutations per site per generation (Ossowski et al. 2010) to calculate the effective population sizes, we get about 2.5×10^5^ individuals, which is within the credibility intervals given in François et al. (2008). Given a generation time of one generation per year, the split between these two southern populations occurred approximately 83,000 years ago. Therefore, according to our estimates, it is unlikely that the ancestors of both populations survived the last glaciation in a common southern refugium as suggested by Sharbel et al. (2000). However, our results for *A. thaliana* are preliminary at best because we have assumed a very simplistic demographic model, *e.g.,* without allowing for population size changes. The per-site population-mutation rate in the *A. thaliana* data set is in a range where our simulations (Fig. S5) indicated a minimal bias of using ISM, rather than FSM, in Jaatha. The simulated data sets were, however, much smaller than the *A. thaliana* data set. Relative to the estimation accuracy that is possible with NGS, the bias introduced using ISM may be large even under conditions for which our simulation studies indicated that the ISM bias was small. Indeed, the ISM-based estimations (Table S4) differ from the FSM-based estimations for the divergence time. For the other parameters, the bias introduced using ISM was minimal.

Because of its computational efficiency, Jaatha has a great potential for population genetic analyses of NGS data. We are currently improving Jaatha's applicability for NGS data by adding procedures to account for sequencing errors and the influence of coverage. To make Jaatha appropriate for genome-wide data, we will allow for variation in mutation rate between loci and the possibility that a certain fraction of the loci are subject to natural selection. In principle, large sequence data sets (with many unlinked or weakly linked loci) should make it possible to fit complex models. To make this feasible in Jaatha, we are extending our approach to allow for more than two populations to be studied and for multiple categories of SNPs, for example, into synonymous, nonsynonymous, and noncoding. This will be necessary to extract more information from the data, which is required to estimate the additional parameters of more complex models. Moreover, to make our bootstrap approach for computing confidence ranges tractable for large data sets, we need highly efficient methods to simulate structured ancestral recombination graphs (Griffiths and Marjoram 1996). We are currently exploring whether McVean and Cardin's (McVean and Cardin 2005) approximation is appropriate or whether we need to account for more of the stochastic dependencies induced by the ARG (Wiuf and Hein 1999). Since composite-likelihood methods require large data sets (Wiuf 2006; Garrigan 2009), we believe Jaatha is a powerful tool in this era of NGS data and has great potential for further applications and extensions.

## Supporting Information

**Data S1.** Parameter ranges and command lines.

**Data S2.** Optimization of Jaatha settings.

**Data S3.** Choice of summary statistics.
