# Supplementary material for: Why to account for finite sites in population genetic studies and how to do this with Jaatha 2.0
Source: Ecol Evol. 2013 Sep 4;3(11):3647–62. doi: 10.1002/ece3.722 (PMC3810865; doi:10.1002/ece3.722)
Supplement: Supplementary file 1 [file ece30003-3647-SD1.pdf]

Online Supplement

S1 Parameter Ranges and Command Lines

S1.1 Basic Demographic model

The data sets were simulated with $nLoci$ loci. The population mutation parameter θ (per locus) and the recombination rate ρ (per locus) were drawn uniformly from the given parameter ranges. The other parameters were chosen uniformly from the following ranges after log transformation:

population-scaled mutation rate $\theta \in [5, 20]$
recombination rate $\rho \in [5, 20]$
size ratios q**,** s_1 **and** $s_2 \in [0.05, 10]$
migration rate $m \in [0.005, 5]$
divergence time $\tau \in [0.017, 20]$

ms command line (Hudson 2002)
`ms 50` $nLoci$ `-t` θ `-r` ρ `1000 -I 2 25 25 -m 1 2` m `-m 2 1` m `-n 2` q `-eN` τ `(`s_1+s_2`)` `-ej` τ `2 1 -g 1` $\frac{log(\frac{1}{s1})}{\tau}$ `- g 2` $\frac{log(\frac{q}{s2})}{\tau}$

S1.2 Decreasing Migration Model

For the "Decreasing Migration“ model, the parameter values for θ, m, ρ, q, s_1, s_2 were chosen as in the basic model (Sect. S1.1). The data sets were simulated with $nLoci$ loci with the following two additional parameters drawn uniformly from the parameter ranges after log transformation:

times τ_m **and** $\tau_0 \in [0.017, 15]$

The divergence time τ is the sum of the time with (τ_m) and without (τ_0) gene flow.
ms command line (Hudson 2002)
`ms 50` $nLoci$ `-t` θ `-r` ρ `1000 -I 2 25 25 -m 1 2 0 -m 2 1 0`
`-em` τ_0 `1 2 (`$0.5 \cdot m$`) -em` τ_0 `2 1 (`$0.5 \cdot m$`) -em (`$0.5{\cdot}\tau_m$+ τ_0`) 1 2` m
`-em (`$0.5{\cdot}\tau_m$+ τ_0`) 2 1` m `-n 2` q `-eN` τ `(`$s1$+s_2`) -ej` τ `2 1 -g 1` $\frac{log(\frac{1}{s_1})}{\tau}$ `-g 2` $\frac{log(\frac{q}{s_2})}{\tau}$

S1.3 Finite-Sites Mutation Models

All parameters were chosen as described in the case of the ”Basic Model“ (Sect. S1.1) with one additional parameter uniformly drawn on the logarithmic scale:

Γ-shape parameter $\alpha \in [0.001, 2.5]$

The `ms` and `seq-gen` command lines for a HKY model are shown for the ”Basic Model“, where L is the sequence length being simulated, T is the factor of the divergence time to the outgroup, ti/tv is the transition transversion ratio, and α the Γ-shape parameter. The base frequencies following the `-f` option were always set to the values observed in the tomato loci. The output of `ms` is a file called ”treeFile“ which serves as an input for `seq-gen`.

ms (Hudson 2002) **and seq-gen** (Rambaut and Grassly 1997) **command lines**

ms (50+1) $nLoci$ -r ρ L -I 3 25 25 1 -m 1 2 m -m 2 1 m -n 2 q -eN τ (s_1+s_2) -ej $T{\star}\tau$ 3 1 -ej τ 2 1 -g 1 $\frac{log(\frac{1}{s_1})}{\tau}$ -g 2 $\frac{log(\frac{q}{s_2})}{\tau}$ -T | tail -n +4 | grep -v // > treeFile

seq-gen -mHKY -l L -s $\frac{\theta}{L}$ -p (L+1) -t ti/tv -f 0.26 0.20 0.22 0.32 -a α < treeFile

As the frequency of back-mutations and double hits not only depends on the average mutation rate but also on the transition-transversion ratio and the heterogeneity of mutation rates across sites, we used the HKY+Γ substitution model (Hasegawa et al. 1985; Yang 1996). For three values of ti/tv (1,2,5), ten sequence files each were simulated with 100 loci and 25 samples per population under the "FixedS2+Γ" model with the *Solanum* base frequencies and $T = 2$ (Jaatha settings `J4` in S1). Parameter values for θ, q, τ, m, and α were uniformly drawn from the log-scaled parameter range given in Section S1.3. We fixed the values of ti/tv in Jaatha to the true ti/tv values with which the data sets were simulated. Only 30 data sets were used in this analysis because including α estimation increases the run time of the sequence simulator. Jaatha was run with the 30 SS described in Section S3 ($n_{SS} = 30$). For a comparison, we also estimated parameters with the ISM with similar settings (`J5` and `J6` in S1).

S2 Optimization of Jaatha Settings

To test the influence of six different Jaatha options (k, s_{ini}, s_{main}, r, ϵ, and w) on the accuracy and the run time, we conducted an analysis in which the two parameters θ and τ were estimated with the following parameters of the basic model fixed to values previously estimated for the tomato data (Naduvilezhath et al. 2011): $s_1 = 1$, $s_2 = 0.3$, $m = 0.5$, $q = 4.5$, and $\rho = 20$ which is the population recombination rate per locus also scaled with $4N_e$. The data sets consisted of 100 loci simulated under an ISM with 25 samples per population. Four values each of τ and of θ were chosen on a uniform grid from the log-transformed parameter range described in Section S1.1. For each of the above mentioned settings three values were tested: $k \in$\{2, 3, 4\}, $s_{ini} \in$\{100, 200, 300\}, $s_{main} \in$\{200, 400, 600\}, $r \in$\{0.05, 0.1, 0.2\}, $\epsilon \in$\{0.5, 1, 2\}, and $w \in$\{0.7, 0.9, 1\}. Each of the 729 ($= 3^6$) program-setting combinations were tested on 16 data sets (one for each θ-τ combination) such that in total 11,664 runs were evaluated. The other Jaatha settings were kept fixed at $n_{SS} = 23$, $t_{stop} = 5$, $n_{loc} = 70$, $n_B = 10$, $ext_\theta = true$, $s_{final} = 200$, $t_{max} = 200$, and $n_{RP} = 10$. The accuracy was measured for each parameter $p \in \{\theta, \tau\}$ in terms of the root mean squared error (RMSE) between the simulated p_{true} and estimated value p_{est}:

$$RMSE(p) = \frac{\sqrt{(p_{est} - p_{true})^2}}{p_{true}} \tag{2}$$

Decreasing the size of the parameter range from which random samples were chosen for the simulations (r) had the greatest influence on precision of the estimates of τ (A 1(a)), but run time increased from an average of 30 minutes for r=2 to 40 minutes for r=0.05 (CPU time on a single kernel of a Quad-Core AMD Opteron with 2.7 GHz). The same effect is captured in a simple linear model in which the run time is the response variable and the different settings the explanatory ones. Decreasing the threshold for the stopping criterion (ϵ) also had a small positive effect, which is barely visible in Figure A 1(b). The number of simulations $s_{ini} \in [100, 300]$ and $s_{main} \in [200, 600]$ in Jaatha showed almost

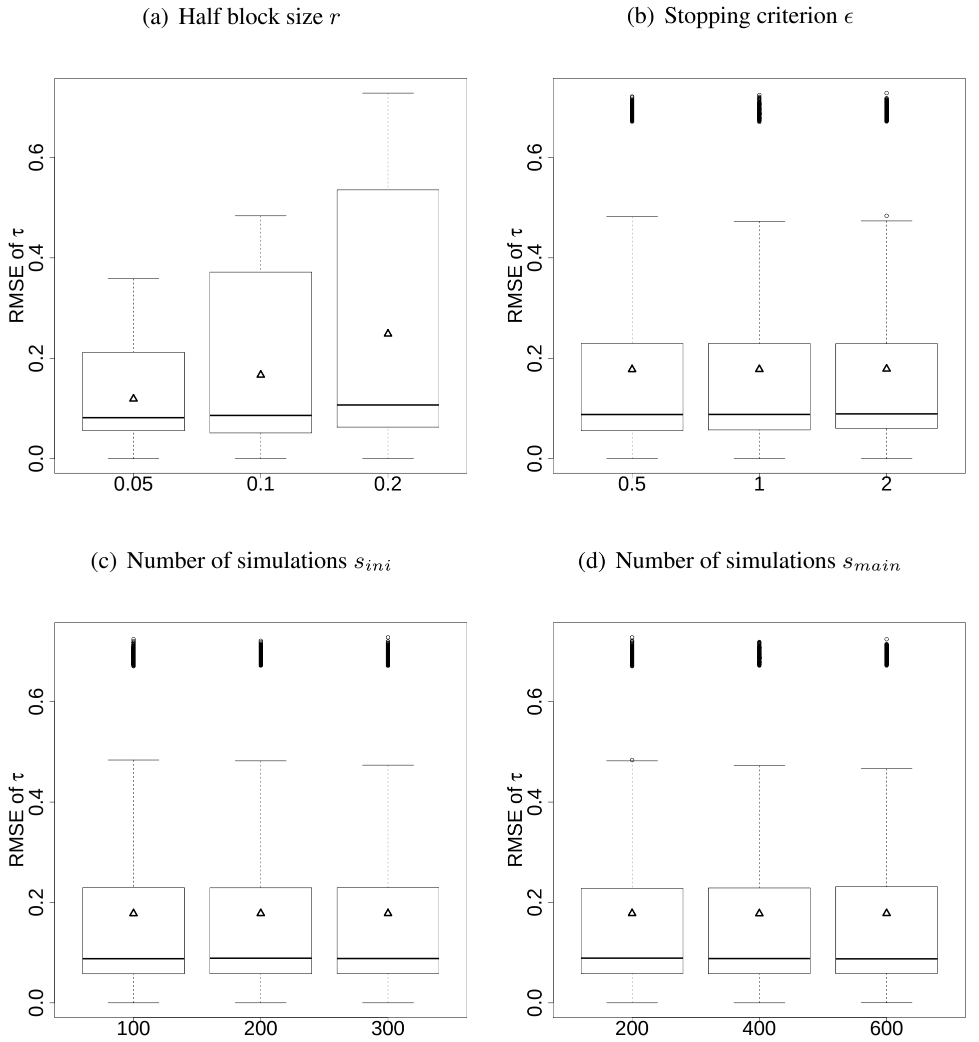

Figure S1: **Influence of Jaatha settings on RMSE of divergence time τ.** The mean RMSE is depicted as $\triangle$. Decreasing the size of the parameter range of the simulation area (r) increases the precision of τ estimation. For the other three settings, little effect on RSME could be observed, although decreasing r or ϵ increases run time (data not shown).

no effect on run time and, surprisingly, on the accuracy in the explored ranges (A 1(c) and A 1(d)). Nevertheless other Jaatha runs show that increasing the number of simulations helps for convergence, and therefore increasing the number of simulations especially in the refined search (s_{main}), is recommended. The RMSE of τ increased drastically as the true divergence time increased (S4). In the two-parameter scenario, decreasing the weights (w) of old simulation blocks or dividing the parameter space into more starting blocks (k) influenced neither the RMSE of the estimates, nor the run time. The effects on the estimation of θ were similar to the ones described for τ although RMSE was lower.

Hence if a fast but accurate search is to be conducted, the following settings are appropriate: $s_{ini} = 100$, $s_{main} = 200$, $r = 0.05$ (or even smaller), $k = 2$ or 3 (depending on the dimension of the parameter range), and $\epsilon = 2$. However, we point out that including additional parameters adds an extra dimension to the parameter space and advise choosing Jaatha settings after a series of trial runs on simulated data.

S3 Choice of Summary Statistics

We define seven additional SS, which are believed to be sensitive for detecting recurrent mutations, and we evaluate whether including these additional SS improves the accuracy ($n_{SS} = 30$). These SS were defined as the number of positions which contained

- S_{24}: three base types in population P_1 or three base types in population P_2
- S_{25}: four base types in population P_1 or four base types in population P_2
- S_{26}: transitions within one population and transversion to outgroup
- S_{27}: transitions in both populations and transversion to outgroup
- S_{28}: transversions within one population and transition to outgroup
- S_{29}: transversions in both populations and transition to outgroup
- SS_{30}: a base present in at least 95% of the samples in one population and in the other population in at most 5% of the samples

The summary statistics SS_{24}-SS_{29} should contain information about the divergence of the two species and SS_{30} about recent migration events. To compare the performance of the 23 original SS $SS_1, \ldots, SS_{23}$ with the extended set $SS_1, \ldots, SS_{30}$ and to decide whether to set the option ext_θ, we simulated 25 genealogies with 100 loci each under the "FixedS2" model and $T = 2$. Sequences of 1 kb in length and with two repetitions were generated using the HKY + Γ model with the *Solanum* base frequencies, $ti/tv = 3$, and $\alpha = 0.7$. The four parameters to estimate were θ, q, m, and τ. The initial search phase however, was only conducted with $n_{SS} = 23$ and for the refined search the same starting points were chosen for the run with $n_{SS} = 23$ and $n_{SS} = 30$. The Jaatha settings `J2` and `J3` with the appropriate n_{SS} were used (Tab. S1).

Additionally, we evaluated whether θ could be calculated proportionally to the number of observed segregating sites (ext_θ =TRUE; see Naduvilezhath et al. 2011) under an FSM, or if θ should be estimated within Jaatha as well (ext_θ =FALSE), hence increasing the number of dimensions and run time. Including θ into the optimization range improved the estimates (cp. in Fig. S2 results marked with "ext" and without). The inclusion of additional SS increased the precision in θ and q estimates only in the case when θ was calculated externally (ext_θ). However, there was no improvement in the estimations of divergence time τ or the migration rate m.

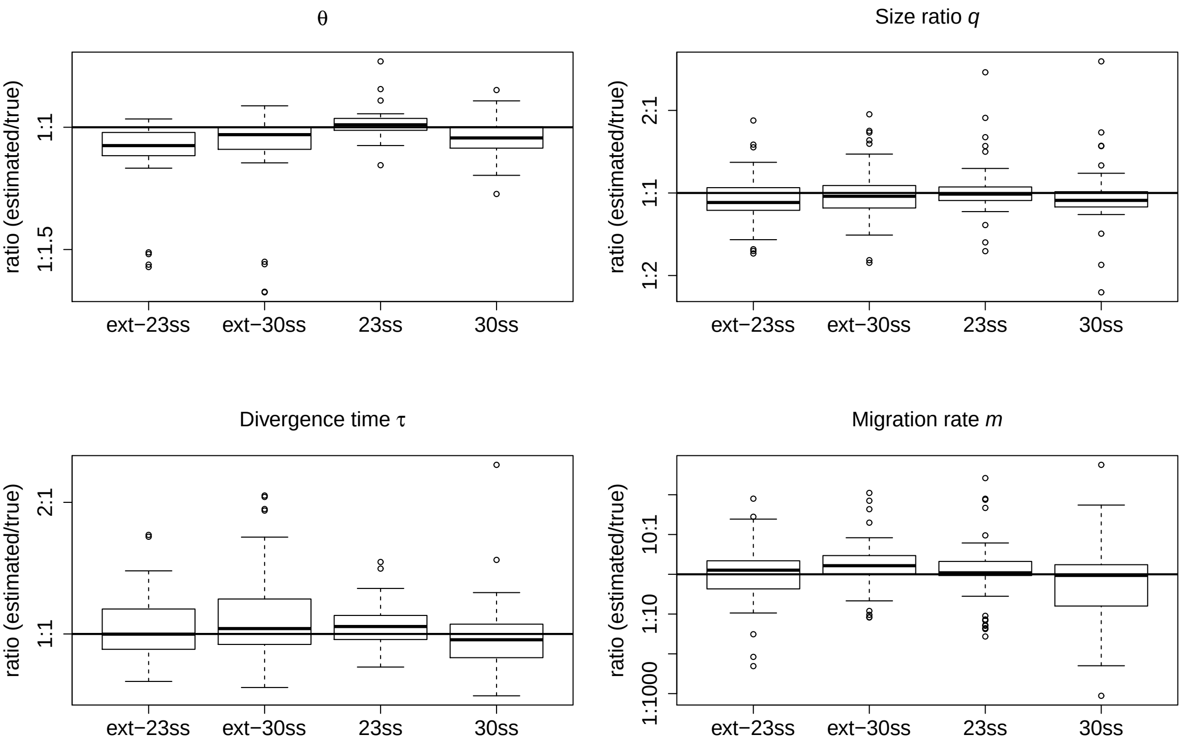

Figure S2: **Comparing different numbers of SS and Jaatha setting ext_θ.** Here we compared the usage of 23 and 30 summary statistics (SS) on the same 25 genealogies (each with two finite-sites sequence simulator runs). Additionally, we assessed the effect of setting the Jaatha option ext_θ, *i.e.* either estimating θ outside of the simulation range (marked with ”ext“; as in Naduvilezhath et al. 2011) or included θ into Jaatha's optimization range. The best overall results were obtained when θ was included in the parameter optimization range and using 23 SS. However, when the option ext_θ was used, including additional SS improved the estimates of θ and q.

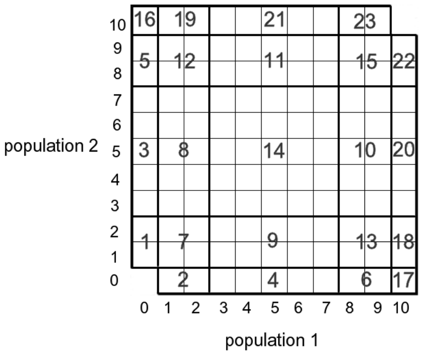

Figure S3: **The 23 summary statistics used in this study for example with $y_1 = y_2 = 10$.** The A_i were further refined for low frequency and high frequency variants whereas middle frequency variants were more coarsely binned. For a general description of the A_i please refer to Naduvilezhath et al. (2011).

S4 Additional Tables and Figures

Table S1: **Jaatha settings** used for the different analyses. The columns stand for the following settings in Jaatha (for more detailed explanation see section describing the new Jaatha version): k- number of (#) intervals each of the n dimensions is divided into (results in k^n start blocks), s_{ini}-# simulations per block in the initial search, s_{main}-# simulations per block in the refined search, r-half side length of the blocks in refined search, ϵ-score difference required for stopping, n_{RP}-# best start points, n_{SS}-# summary statistics, ext_θ-`true`: θ is calculated outside of the block during refined search and -`false`: θ is calculated like the other parameters, M_{ini}-mutation model for initial search, M_{main}-mutation model for refined search, s_{final}-# simulations for the calculation of likelihoods, t_{max}-maximum # steps during refined search, $scale$-only $\frac{1}{scale}$ of the loci are simulated and the JSFS is extrapolated accordingly. Weight w was always kept at 0.9, # steps t_{stop} in which there was no score change of at least ϵ at 5, # simulated loci n_{loc} for the GLM fittings at 70, and # best parameter combinations n_B kept in each $\mathcal{L}$ list at 10.

Reference	k	s_{ini}	s_{main}	r	ϵ	n_{RP}	n_{SS}	ext_θ	M_{ini}	M_{main}	s_{final}	t_{max}	$scale$
J1	3	200	200	0.05	1	10/16	23	TRUE	IS	IS	200	200	1
J2	3	100	200	0.05	2	4	23/30	TRUE	FS	FS	100	200	1
J3	3	300	200	0.05	2	10	23/30	FALSE	FS	FS	100	170	1
J4	2	300	200	0.1	2	10	23	FALSE	FS	FS	100	170	1
J5	2	300	200	0.1	2	8	23	FALSE	IS	IS	100	170	1
J6	3	300	400	0.1	2	10	23	FALSE	IS	IS	100	170	1
J7	3	300	200	0.1	2	16/10	23	FALSE	FS	FS	200	170	1
J8	3	300	200	0.1	2	9	23	FALSE	IS	FS	200	170	1
J9	3	300	200	0.05	2	16	23	FALSE	IS	FS	100	170	1
J10	3	300	200	0.05	2	16	23	FALSE	IS	IS	200	170	1
J11	3	300	200	0.1	2	16	23	FALSE	IS	FS	200	170	1
J12	3	200	300	0.05	1	16	23	FALSE	IS	FS	300	170	1
J13	2	400	300	0.1	2	16	23	FALSE	FS	FS	300	170	1
J14	3	200	500	0.05	0.5	40	23	TRUE	IS	IS	200	200	1
J15	2	300	200	0.1	2	16	23	FALSE	FS	FS	100	170	1
J16	3	400	200	0.1	2	16	23	FALSE	IS	FS	300	170	1
J17	3	200	200	0.05	50	4	23	TRUE	FS	FS	200	200	250
J18	3	200	200	0.05	25	4	23	TRUE	ISM	ISM	200	200	250

Table S2: **Estimated parameter values for the seven *S. peruvianum* and *S. chilense* loci with alternative settings under three different models.** θ_{site}, m and τ are scaled with $4N_1$, where N_1 is the effective population size of *S. chilense*. Bold values are fixed parameter values for the estimation. The corresponding Jaatha settings can be found in S1.

**Model**	θ_{site}	q	m	τ	s_1	s_2	α	# **Parameters**	**log-Likelihood**	**Settings**
(IS) FixedS2	0.010	4.98	0.59	0.38	**1**	0.29	-	5	$-\infty$	`J14`
FixedS2+Γ	0.010	7.07	0.35	0.26	**1**	**0.3**	2.5	5	-76.2	`J15`
BothGrowMig	0.010	4.23	0.73	0.57	0.41	0.05	**0.7**	6	-97.7	`J16`

Table S3: **Jaatha settings and run times** for *Solanum* analyses. The CPU time on a single processor Quad-Core AMD Opteron kernel is reported.

**Model**	**Settings**	**Run time** [h]
NoMig	`J8`	35
NoMig+Γ	`J7`	109
FixedS2	`J9`	83
(IS) FixedS2	`J10`	2
FixedS2+Γ	`J7`	186
SingleGrowMig	`J11`	84
SingleGrowMig+Γ	`J7`	386
BothGrowNoMig	`J11`	35
BothGrowNoMig+Γ	`J7`	322
BothGrowMig	`J12`	105
BothGrowMig	`J16`	72
BothGrowMig+Γ	`J13`	194
DecMig	`J9`	101
(IS) DecMig	`J1`	19

Table S4: **Parameter estimates for *A. thaliana* using ISM.** Jaatha's estimates using the ISM for the mutation rate θ, time τ of the split of both demes, the subsequent migration rate m between populations, and the rate heterogeneity parameter α. The parameter τ is scaled in $2N_e$ generations, m is twice the number of immigrants to each deme per generation, and θ is $2N_e$ times the mutation rate per base.

	τ	m	θ_{site}
complete dataset	0.27	3.17	$3.23 \cdot 10^{-3}$
FS only	0.24	3.59	$2.40 \cdot 10^{-3}$
Th only	0.24	3.61	$3.56 \cdot 10^{-3}$
NC only	0.22	3.12	$4.07 \cdot 10^{-3}$

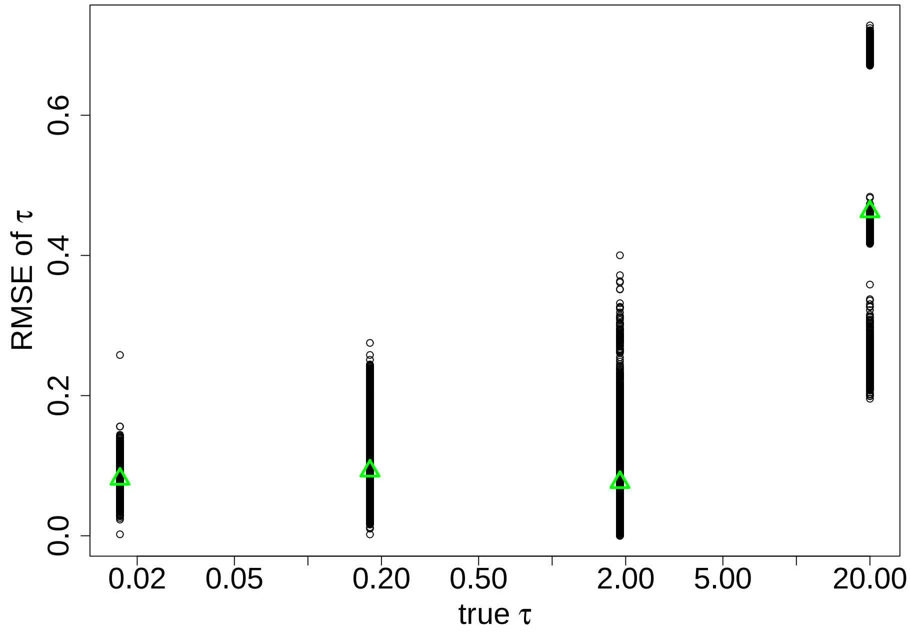

Figure S4: **Jaatha becomes imprecise when estimating large divergence times** ($\tau = 20$)**.** The true value of the divergence time τ is plotted against the RMSE of τ ($\circ$). The average value is shown as $\triangle$. As τ gets larger Jaatha has trouble estimating the correct value of τ.

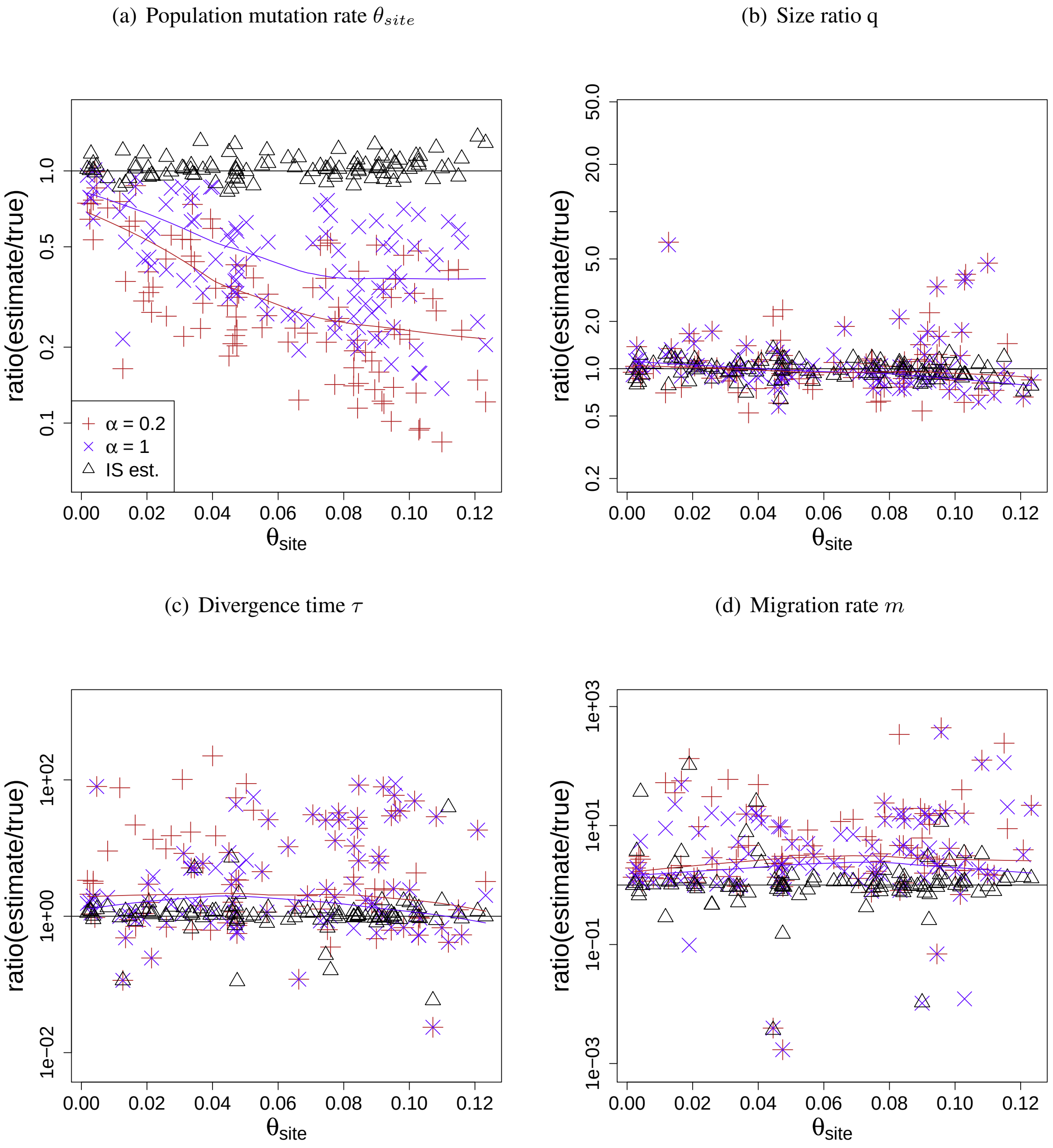

Figure S5: **The effect of neglecting finite sites on parameter estimation under the "Constant" model.** The ratio of estimated and true values of θ, q, τ, and m plotted against true θ values under infinite-sites assumptions and the "Constant" model. Shown are the data sets simulated with the most extreme α values ($\alpha = 0.2$ and 1), $ti/tv = 2$, $T = 3$. As a comparison, estimates for infinite-sites data sets ($\triangle$) are included. The lines plotted are polynomial regression lines fitted to the ratios (with *lowess* function of R).

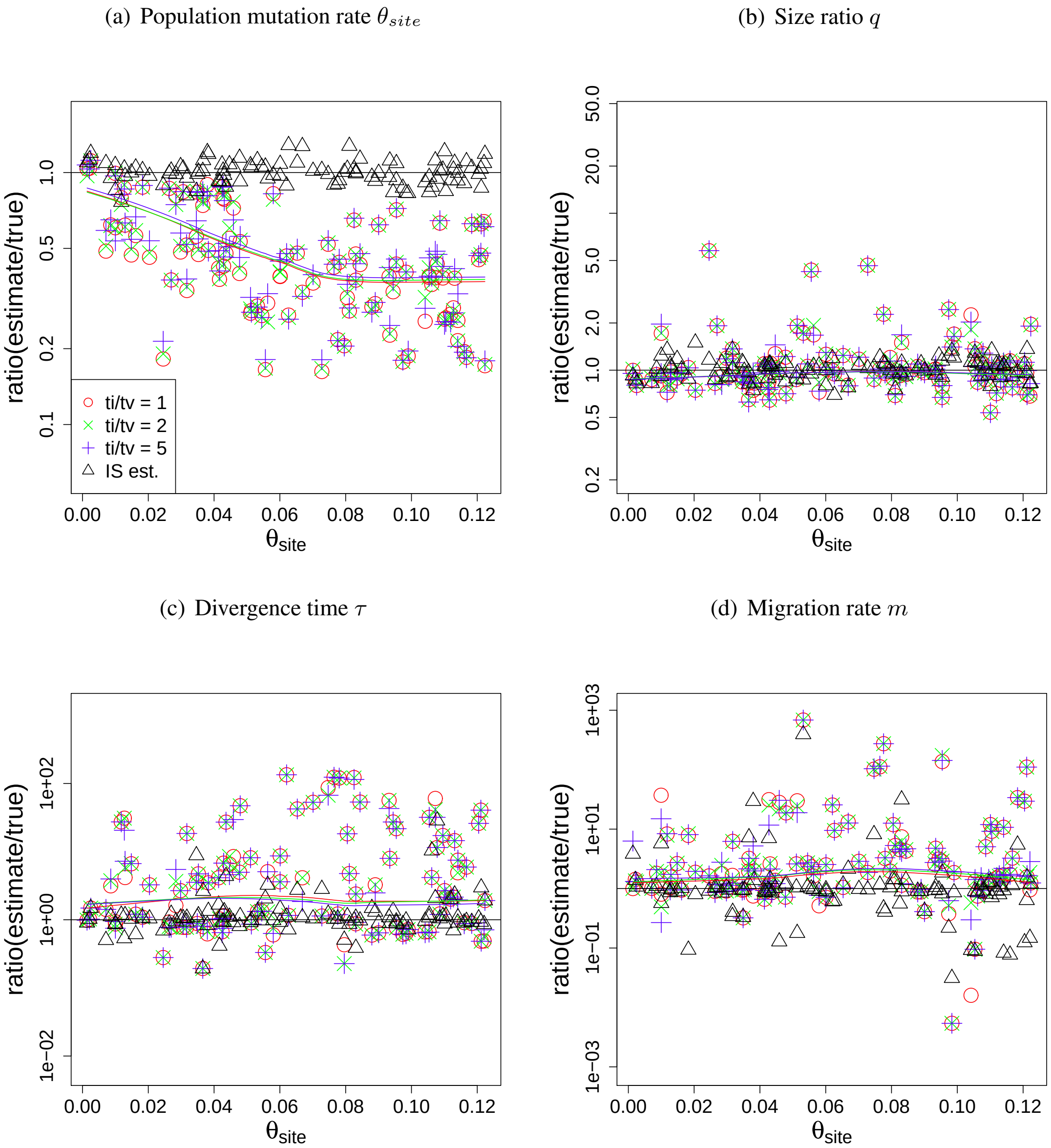

Figure S6: **Different transition-transversion ratios have almost no influence on the estimations.** The ratio of estimated and true values of θ, q, τ, and m plotted against the true θ values under infinite-sites assumptions for three different values of ti/tv (1, 2, 5). The data were simulated with a finite-sites model with $\alpha = 1$ and $T = 6$ under the "Constant“ model. As a comparison, estimates for infinite-sites data sets (△) are included. The lines plotted are polynomial regression lines fitted to the ratios (with *lowess* function of R).
